# Vocal learning in animals and humans

**DOI:** 10.1098/rstb.2020.0234

**Published:** 2021-10-25

**Authors:** Sonja C. Vernes, Vincent M. Janik, W. Tecumseh Fitch, Peter J. B. Slater

**Affiliations:** ^1^ School of Biology, The University of St Andrews, St Andrews, UK; ^2^ Neurogenetics of Vocal Communication Group, Max Planck Institute for Psycholinguistics, Nijmegen, The Netherlands; ^3^ Donders Institute for Brain, Cognition and Behaviour, Nijmegen, The Netherlands; ^4^ University of Vienna, Vienna, Austria

## 1. Introduction

That some animals can learn the sounds they produce has been known for millennia [1], most notably through the ability of birds like parrots to copy human speech. But just how widespread vocal learning is, and how it impacts on the natural lives of animals that show it, is a subject that has only been illuminated relatively recently and continues to raise interesting and challenging questions, many of them investigated by articles in this issue.

**Figure 1.**
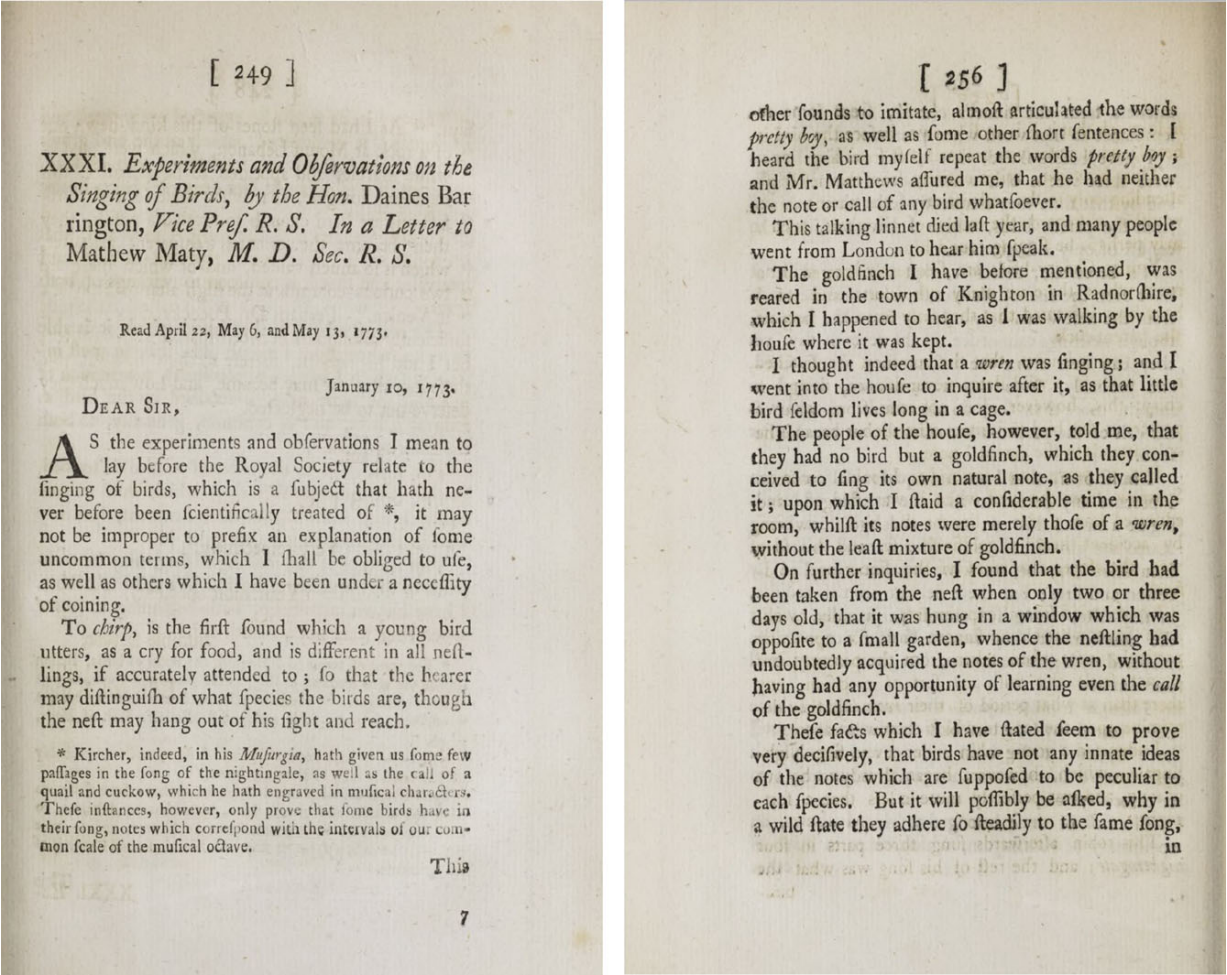
Experiments and observations on the singing of birds, by the Hon Daines Barrington, Vice President of the Royal Society. January 10, 1773, pp. 249, 256 [2].

Almost 250 years ago, in a paper published in this very journal, observations by Daines Barrington (then Vice President of the Royal Society) raised such questions (figure 1) [2]. However, it was not at that stage possible to get far towards answering them. Barrington was writing before the birth of Darwin and so knew nothing of the major theoretical framework within which much of current biological research is conducted. Despite this, he had an insightful view of the capabilities of at least some bird species, pointing out that ‘Notes in birds are no more innate, than language is in man, and depend entirely upon the matter under which they are bred, as far as their organs will enable them to imitate the sounds which they have frequent opportunities of hearing’ ([2], p. 252).

Wrens and goldfinches look very different and their songs are extremely distinctive so a good observer such as Barrington had no difficulty in concluding that one of the latter had learnt the song of the former (see Page 256 in figure 1). Moving on from this discovery to looking at the role of this learning ability in the natural lives of animals needed later technical advances. Ways of recording sounds and replaying them lay at least a century and a half in the future. Indeed, even then, these were extremely cumbersome and far from easy to use. Thorpe [3] carried out one of the first experimental studies of vocal learning and, in addition to using the tape recorders which had recently been developed, he also recorded the songs of his chaffinches with a stylus on lacquer-coated discs. He found this in some ways preferable as one could see where the needle had registered a sound on the disc without going right through the recording to find it. Thorpe was also the first to use the sound spectrograph, which revolutionized sound analysis in the mid-twentieth century. Now, of course, such studies no longer require committed expensive pieces of equipment like spectrographs but can be carried out on a personal computer such as we all have on our desks; nor do we need tape recorders when the sounds can be registered directly on hard drives.

That many people have become fascinated by vocal learning is at least partly because it is a striking feature of our own species, and one fundamental to its success. Yet it is also one for which evidence in our closest relatives has so far proved elusive. While research into birds has continued to lead the field, in recent years studies in mammalian species have increased and enabled more cross taxa comparisons [4]. Studying vocal learning in a wide range of animals has the potential to shed light on why it arose so extraordinarily productively in our own recent evolution [5]. The research papers and reviews in this issue illustrate the many different approaches that can be taken to vocal learning across diverse species. This collection presents a mix of review and theory papers, alongside empirical research papers concerned with experimental and modelling approaches to the study of vocal learning. We have sought to include papers that address or discuss all of the groups in which the trait has been identified, including oscine and non-oscine birds, cetaceans, pinnipeds, bats, elephants and humans.

## 2. Frameworks for vocal learning across species

In this Introduction, we shall attempt to draw together some of these threads, starting with several articles that look at the phenomenon in a broad context and across a wide variety of species to develop frameworks for study. The expression ‘vocal learning’ has in the past been used simply to refer to the use by one animal of sounds copied from others. However, it has become clear in the last couple of decades that learning impinges on the use of sounds by animals in numerous ways, of which this is only one, and a number of suggestions have been made on how these should be classified during that time [6–9]. Vernes *et al.* [10] review and discuss these to come up with suggestions as to how the phenomena involved should be defined and identified. This makes it clear that the issues involved are far more complex than whether or not a particular sound is learnt and their discussion will provide a useful detailed framework to guide research in the years to come. Carouso-Peck *et al.* [11] ask what selective advantages contributed to the evolution of vocal learning when many species appear to solve similar problems without this skill. They highlight how vocal learning research has focussed mostly on songbirds in the context of sexual selection and aim to draw attention to other vocal learners and other contexts in which this skill is being used. Using humans and parrots as examples, they review what these advantages might be and conclude that increased repertoire sizes improve the efficacy of signals and/or allow for flexible pruning to arrive at optimized signals. They also hypothesize that vocal learning may have evolved because of the advantages of cumulative vocal culture. A review by Searcy *et al*. [12] emphasizes the diversity of types of vocal learning in the 4000+ species of songbirds. Although all songbirds are thought to be vocal production learners, they vary considerably in the degree to which they depend upon external input, and some songbirds such as sedge warblers can develop apparently normal songs even lacking such input. The degree to which deafening of young birds leads to aberrant song also varies between species, suggesting that the need for auditory-motor feedback also is variable. After concisely reviewing these factors, the authors propose a taxonomy of vocal production learning in birds that allows for degrees of vocal learning, depending upon the specific conditions required to develop normal vocalizations. Lattenkamp *et al.* [13] provide ‘A researcher's guide to the comparative assessment of vocal production learning’, that considers how vocal learning abilities are experimentally assessed and reported in different studies, and across species. They highlight discrepancies in reporting, suggest ways in which reporting could be made more uniform, and propose five factors that would increase comparability of data across studies. The guidelines presented therein would facilitate future meta-analyses and comparative studies if widely adopted, enriching our understanding of vocal learning.

## 3. Distribution of vocal learning in birds and mammals

Moving on from these general considerations, several articles examine the distribution of vocal learning in birds and mammals, the only two classes in which it has so far been demonstrated. In addition to parrots and songbirds, evidence for vocal production learning has recently come to light in a number of other bird groups, showing that its occurrence is now more widespread than realized even a few years ago. ten Cate [14] reviews this evidence in non-oscine birds, evaluating the evidence and highlighting unusual examples from across this group. This suggests a number of species that deserve further study and gives new insight into the possible evolution of vocal learning in this group. A particularly surprising finding, described in detail for the first time, to our knowledge, by ten Cate & Fullagar [15], is clear evidence for production learning in the Australian musk duck (*Biziura lobata*). The musk duck belongs to a basal clade in avian phylogeny and no other Anseriform species has been described as showing vocal learning. The musk duck is unusual in that it is altricial and also shows lekking behaviour, but just why it evolved vocal learning must await detailed field studies. These two papers support the hypothesis that vocal learning has evolved independently several times among the birds, rather than evolving once and being lost many times.

Rather few species have been described in which social guidance is involved in influencing vocal learning, most obviously humans and, among songbirds, brown-headed cowbirds [16]. But this may stem from the subtlety of the interactions involved so that the possibility has not been examined in many. Carouso-Peck & Goldstein [17] have looked at a wide variety of songbirds and identified several features that make such influences likely. They thus pinpoint a group of species, scattered across the phylogenetic tree of songbirds that deserve study from this viewpoint.

In 1997, Janik & Slater [18] published a highly cited review of mammalian vocal learning. Now, almost 25 years on, Janik & Knörnschild [19] revisit and update the evidence for vocal learning across mammals, dealing in particular with those non-human mammals where the evidence is clear: cetaceans, pinnipeds, elephants and bats. The abilities of other mammals are also considered, including primates, mole-rats, goats and mice. The authors emphasize the benefits of increasing numbers of studies addressing vocal learning in mammals, and suggest that addressing variability in learning skills between these species is a key issue for the future.

Dolphins present one of the most well-established examples of mammalian vocal learning. Famously, dolphins use learned calls, known as signature whistles, in the context of individual recognition [20]. Oswald *et al.* [21] investigated species differences in whistle contours of two closely related, sympatric species of common dolphin, *Delphinus delphis* and *Delphinus bairdii*. Using a large number of acoustic recordings from the wild, the authors identified distinctive whistle modulation patterns that each species uses. They suggest that these differences may facilitate species recognition and be culturally driven—an interesting area for future vocal learning studies in dolphins.

Following initial reports of production learning, Stoeger & Baotic [22] present to our knowledge, the first evidence for vocal usage learning in African and Asian elephants, reporting results from training sessions in which the animals vocalize in response to trainer cues. Several of the animals were able to give different vocalizations in response to different cues, and some developed novel vocalizations in this paradigm. This adds to the evidence that usage learning is widespread in mammals and birds.

## 4. Vocal learning early in life

A continuing interest in vocal learning concerns precisely when during ontogeny animals learn the sounds they produce, with evidence from some of a very restricted sensitive period and in others of learning throughout life. Several papers here examine learning early in life. It is often assumed that the sensitive period for vocal learning in birds commences sometime after hatching. However, as the paper by Colombelli-Negrel *et al.* [23] makes clear, embryos of various species can perceive and respond to sounds *in ovo*. The species in the study are few, but highly varied, including a Darwin's finch, two fairy wrens, a penguin and a quail. All show habituation in heart rate response to repeated conspecific sounds heard in the egg. There was a reduction of heart rate in two vocal learning species played calls of their own or of another species, whereas two non-learners only responded to their own—an interesting glimpse of what may turn out to be an important widespread difference when tested on a greater number of species. The results point to an area ripe for further exploration.

Zebra finches have long been a preferred species for laboratory studies of vocal learning and are known to learn their songs early in life. If housed with their parents, young males learn precise copies of their father's song, often including the exact sequence of the elements involved. The study by Mol *et al.* [24] comes up with the interesting, and somewhat surprising, result that recognition of the father's song persists despite various manipulations of its sequence. Thus, while birds clearly code sequential information when they learn their fathers' songs, syllable sequence does not appear important for its subsequent recognition.

Among mammals, pinnipeds have long been thought capable of vocal learning, based on the remarkable case of ‘Hoover’, a harbour seal that convincingly imitated human speech [25], but controlled experimental data have been lacking until recently [26], and the relevance of vocal learning in pinnipeds' own vocal communication has remained unclear. Stansbury & Janik [27] report, to our knowledge, the first experimental field study on this topic, involving playbacks to wild grey seals at a breeding colony. They played modified seal sounds to eight seal pups, and found both that the target pups copied the structure of the synthetic pup calls, and their copies became more accurate over time. Although it remains unclear why pups should copy each other, these results suggest that well-documented regional differences in pinniped vocalizations (dialects) may result from horizontal transfer (among same-aged individuals) and not just the more standard vertical transfer (from adults to young) typical in birdsong.

The development of social calls of the pale spear-nosed bat was investigated by Lattenkamp *et al*. [28] observing the effects of permanent deafening. The strongest effects were on call activity and call duration with deafened bats calling much more but producing much shorter calls than the hearing control group. The most pronounced differences were found in early development, but some differences remained later in life. Deafening experiments lead to a variety of changes in an animal's life including less social input and potentially increased stress levels, which can make interpretations difficult. However, the experiment clearly highlights the importance of vocal input on bat vocal behaviour.

Babbling is another developmental phenomenon, prominent in human vocal learning, that is also found in most vocal learning species. As such it has been hypothesized to be a pre-requisite for vocal learning [29]. ter Haar *et al.* [30] explore evidence for and against this theory, considering babbling from a cross-species perspective. The paper compares the phenotype and potential function of babbling in humans, birds and mammals. Enumeration of the open questions around this topic points towards future research that may help to resolve the link between vocal learning and babbling, the function of babbling, and its mechanistic underpinnings. Oller *et al.* focus on human early vocal development in infants across the first 12 months of life [31]. They present quantitative data on the occurrence of three categories of sounds in the utterances of children; cries/screams, laughter and protophones (potentially communicative vocalizations and babbling). These data demonstrate the predominance of protophones in the repertoire of infants, as their occurrences far outweighed those of utterances that fall into the other categories during this period. Frequent protophone use was observed regardless of the presence or absence of adult produced vocalizations in the recording context. These papers lay a foundation for cross-species exploration of early vocal development, which may aid in understanding evolutionary processes that drove language acquisition.

## 5. Neurobiology of vocal learning

The neurobiology of vocal learning is well studied in some songbirds, such as zebra finches [32]. However, in many bird and mammal species, because of the complexity of the question under study and behaviour involved, the neurscientific basis of vocal learning remains unknown. Hoeksema *et al.* [33] provide detailed information on the fine structure of the grey seal brain. This neuroanatomical atlas reveals that grey seals have a comparatively large temporal lobe and cerebellum, and that the cortex is similar to that in humans in thickness and shows the expected six-layered mammalian structure. The study also found expression of the vocal learning-related *FoxP2* gene in the cortex. The atlas is a valuable basis for further studies on the mechanisms of vocal learning in grey seals.

Humans are of course the most-studied vocal learners of all, and the brain networks underlying our vocal abilities have been studied intensively in the 150 years since Paul Broca identified his eponymous brain area [34]. Advances in brain imaging have consistently enlarged the number of brain areas involved in speech since that time, and the offering by Valeriani & Simonyan [35] applies state-of-the-art connectivity analyses to continue this trend. They examine the causal directionality of links in the widely distributed brain connectivity network underlying coherent speech, focusing on causal (rather than correlational) connections. They find that this network reorganizes dynamically to support the increased complexity of speech production, versus simple repetition of nonsense syllables.

## 6. Computational and modelling approaches to vocal learning

Various avenues of research also arise from recent developments in computation and in modelling techniques opening up possibilities ranging from phylogenetic studies across a wide variety of species to computer simulations of how vocalizations change with time. Regarding phylogeny, Arato & Fitch [36] make use of well-established avian molecular phylogenies to look at the phylogenetic signal of song learning involving both vocal learning and non-learning species. They find that the phylogenetic signal can be as great, or even greater, for learnt songs as for unlearnt ones. Zandberg *et al.* [37] present cultural evolutionary simulations of humpback whale song transmission based on empirical data collected over 10 years in the Southern Hemisphere population. Using observed values for interactions between animals in the Southern and Northern Hemispheres, they simulated the observed patterns of song evolution in both hemispheres. They found that differences in population number and size and the pattern of interactions are sufficient to lead to the difference in speed of song change observed between the North and the South. ter Haar *et al.* [30] also include a consideration of how modelling approaches can be used to test mechanistic theories of babbling behaviour across vocal learning animals.

We hope that the current issue illustrates the vibrant and wide-ranging research approaches currently surrounding the topic of vocal learning. The increasing diversity of perspectives, methods and study species suggests that this field will continue to grow and innovate in the future, leading to exciting new avenues and a clearer understanding of vocal learning and its evolution. We have no doubt that Daines Barrington would be fascinated by how the subject has developed since his time.

## Data accessibility

This article has no additional data.
